# Post-COVID-19 Outbreak of Severe Kawasaki-like Multisystem Inflammatory Syndrome in Children

**DOI:** 10.21315/mjms2021.28.1.14

**Published:** 2021-02-24

**Authors:** Sadia Shakeel, Mohamed Azmi Ahmad Hassali

**Affiliations:** 1 Discipline of Social and Administrative Pharmacy, School of Pharmaceutical Sciences, Universiti Sains Malaysia, Pulau Pinang, Malaysia; 2 Faculty of Pharmaceutical Sciences, Dow University of Health Sciences, Karachi, Pakistan

**Keywords:** multisystem inflammatory syndrome, COVID-19, rare disease, children

## Abstract

With the continuation of the second wave of a novel coronavirus disease (COVID-19), which is likely to be even more devastating, there are several associated health problems. COVID-19 is usually mild and non-fatal in children. However, in rare cases, children could severely be affected, and clinical manifestations may differ from adults. A multisystem inflammatory syndrome in children (MIS-C) is a rare but serious complication associated with COVID-19, initiated by an overactive immune response in kids that usually hits weeks after exposure to the COVID-19. MIS-C is a disorder in which inflammation could occur in different parts of the body. The disease puts pressure on the heart, as blood vessels leading towards the heart get inflamed and incapable of carrying adequate blood, hence producing cardiac complications in children hospitalised with MIS-C. The problem seems to be associated with COVID-19 in children; however, the association between MIS-C and COVID-19 is still unidentified. There is very little understanding of what triggers the MIS-C, which necessitates a rigorous mapping of the disease and associated risk elements for better disease management and navigating through this crisis.

## Introduction

Children and younger persons make up a smaller percentage of coronavirus disease 2019 (COVID-19) sufferers, and the pediatric cases account for only 2.1%–7.8% of confirmed cases in Europe, North America and Asia (1). COVID-19 is usually mild and non-fatal in children and clinical manifestations may differ from adults. In April 2020, reports from the United Kingdom documented a clinical presentation in children identical to Kawasaki disease (KD) or toxic shock syndrome. Since then, there has been an increase in reported cases of similarly affected children in other parts of the world (2). The cases have been reported from Asia, North America, Latin America and Europe in the past 3 months, describing children with COVID-19-associated multisystem inflammatory syndrome in children (MIS-C), which is likely to develop after the disease instead of developing during the acute phase of COVID-19 (3).

KD is a self-limiting and acute vasculitis of the medium calibre vessels. The syndrome originates its name from Tomisaku Kawasaki, a Japanese pediatrician, who described the first case of KD in a 4 years old boy (4). The average age of those suffering from KD is 2 years old and 75% are under 5 years old; boys develop the disease 1.5 times more often than girls (5). The initial sign includes fever, abdominal pain, red eyes, vomiting or diarrhea and a rash on the trunk. Some children may have a swollen red mouth and red tongue, whereas others may have swollen glands in the neck (5, 6).

## Pathophysiology

The pathophysiology of MIS-C is not well recognised. The condition may be due to an abnormal immune response to the virus, with some similarities to KD, macrophage activation syndrome (MAS) and cytokine release syndrome. A post-infectious process could be suggested based on the timing of the rise of these cases relative to the peak time of COVID-19 cases in communities (6).

**Figure 1:**
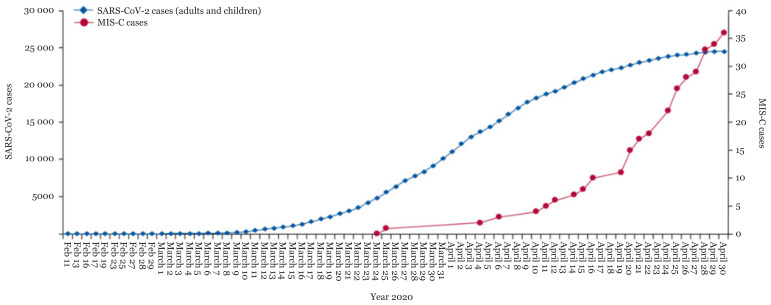
Time passage of MIS-C in PCR-positive COVID-19 cases (8)

The negative polymerase chain reaction (PCR) results for the severe acute respiratory syndrome coronavirus 2 (SARS-CoV-2), but positive serology has been reported in some affected children, a finding that further supports the hypothesis that MIS-C is related to immune dysregulation occurring after the acute infection has passed. According to data from Public Health England, the number of MIS-C cases increased drastically around 16 April 2020, almost 4 weeks after the substantial increase in COVID-19 cases in the UK (8). In the available case series, there were 364 children in whom both PCR and serology were performed. Among them, 58% had positive serology with negative PCR, 30% were positive on both tests and 11% were negative on both tests (7) (Figure 1).

**Figure 2:**
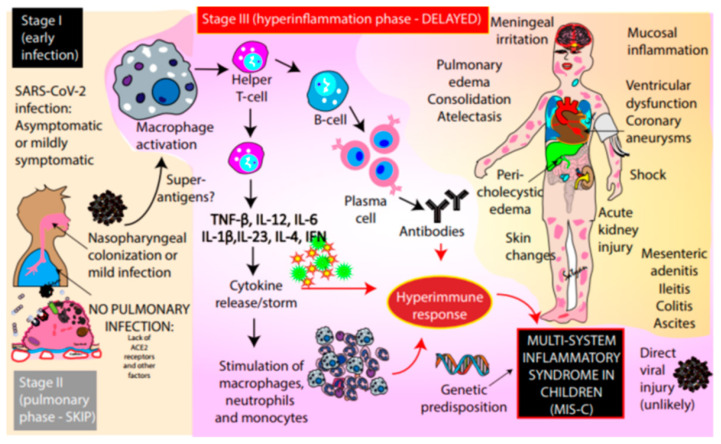
Probable mechanisms of inflammatory processes for MIS-C (13)

Given the resemblances among the adult hyper-inflammatory response and MIS-C, antibodies might contribute to both disorders (9). However, the mechanism of the exaggerated immune response in MIS-C is currently in phase of investigation (10). Antibodies to SARS-CoV might produce the infection through an antibody-dependent increase in viral entrance and replication, as detected in dengue, or by stimulating a host inflammatory response through the formation of immune complexes or cellular stimulation or direct stimulation of anti-tissue antibody or both (11). Identical mechanisms might be involved in the SARS-CoV-2 related inflammatory disorder. SARS-CoV-2 has not been reported in MIS-C patients; therefore, the antibody-dependent inflammation is probable to occur by an acquired immune response instead of an increase in viral replication (8) (Figure 2). Anti-spike antibodies produced against SARS-CoV have been revealing to bring out inflammation in human and in primate macrophages. Thus, the anti-spike antibodies formed against SARS-CoV-2 might perhaps cause inflammation by the same process. Another study reported that immune complexes produced by linking the patient’s anti-spike antibodies with spike protein become the reason for the activation of macrophages that supported the anticipated mechanism for SARS-CoV-2 (12).

The inflammatory conditions activated by SARS-CoV-2 have the same characteristics as KD and might produce coronary aneurysms. Immune complexes have been well recognised in KD and could be the source of mediating vascular injury by triggering inflammatory reactions through the activation of Fc-γ receptor or complement (14). The generation of T-cell responses to SARS-CoV-2 may perhaps likewise contribute significantly to damaging the organs and process of inflammation as increased responses of T-cell were observed in KD (15).

## Diagnosis and Clinical Presentation

**Figure 3:**
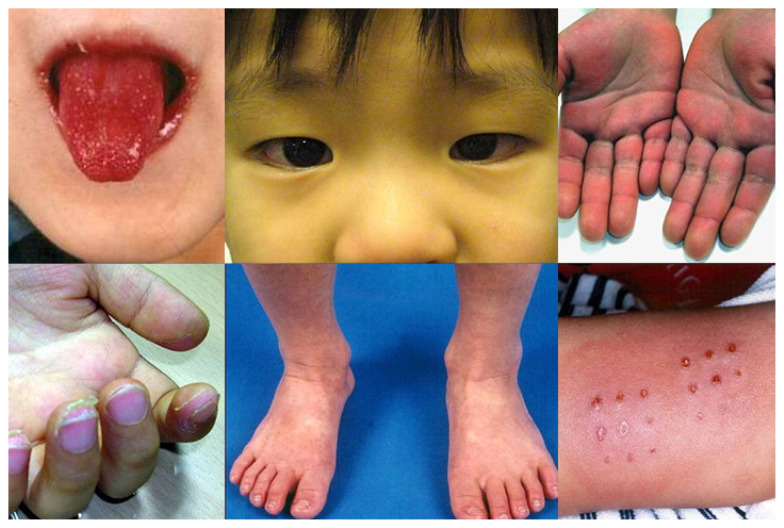
Clinical presentations of Kawasaki-like MIS-C (17)

The MIS-C diagnostic criteria have been recommended by the Centres for Disease Control and Prevention (CDC). It is advised that all healthcare professionals must notify the respective health authorities about the suspected case if they found; a person less than 21 years of age suffering from fever (≥ 38 °C for the duration of ≥ 24 h), laboratory confirmation of inflammation (elevated erythrocyte sedimentation rate, C-reactive protein, procalcitonin, fibrinogen, D-dimer, ferritin, interleukin 6, lactic acid dehydrogenase, elevated neutrophils, lower count of lymphocytes and albumin), individuals with the involvement of (≥ 2) multisystem organ (e.g. respiratory, neurological, haematologic, renal, gastrointestinal, cardiac, dermatological etc.), showing no alternative probable diagnosis and depicting positive indications for recent or current COVID-19. The typical clinical presentations include persistent fevers (median duration 4 days), gastrointestinal symptoms (abdominal pain, vomiting, diarrhea), conjunctivitis, the involvement of mucous membrane, rash, respiratory symptoms, neurocognitive symptoms (headache, lethargy, confusion), swollen hands/feet and sore throat (16) (Figure 3).

## Management

MIS-C is a multisystem illness and care for affected children requires harmonisation of many different specialties. The level of care is determined by the severity of the disease; patients depicting moderate to severe symptoms and signs must be admitted to the hospital. A pediatric intensive care unit admission is suitable for children having hemodynamic instability (arrhythmia, shock), major respiratory compromise, or any other possibly fatal complications (18).

The patients presenting with severe multisystem involvement, particularly those with shock, should receive prompt empiric broad-spectrum antibiotic therapy that consists of ceftriaxone plus vancomycin. Ceftaroline plus piperacillin-tazobactam is an alternative regimen, principally for children with acute kidney injury (19). The role of antiviral therapies (e.g. remdesivir) in the management of MIS-C is uncertain and their use is generally limited to children with severe MIS-C manifestations (20). Additional therapy can be used depending on individual clinical presentation. For example, patients presenting KD with associated distributive shock should receive treatment for KD (i.e., intravenous immune globulin [IVIG] and aspirin) and appropriate haemodynamic support (i.e., volume expansion and epinephrine). The risks and benefits of adjunctive therapies (glucocorticoids, interleukin-1 [IL-1] inhibitors [e.g. canakinumab, anakinra], IL-6 inhibitors [e.g. tocilizumab], convalescent plasma from recovered COVID-19 patients) are uncertain. The use of adjunctive therapies are suggested on a case-by-case basis, according to disease severity and markers of inflammation or active SARS-CoV-2 infection (20, 21).

## Epidemiology

Several cases of this rare disorder have been reported across the world. The initial reports of MIS-C emerged from the UK in April 2020. Since then, there have been reports of similarly affected children in other parts of the world, including Europe, Canada, and the United States. Notably, there have been no reports of MIS-C from China or other Asian countries with high rates of COVID-19 early in the pandemic (22). Many children with MIS-C meet the criteria for complete or incomplete KD. However, the epidemiology differs from that of classic KD. Most MIS-C cases have been reported in older children and adolescents who were previously healthy. By contrast, classic KD typically affects infants and young children and has a higher incidence in East Asia and children of Asian descent. The epidemiology of MIS-C also differs from that of acute COVID-19 illness in children, which tends to be most severe in infants < 1 year of age and in children with underlying health problems. The first report of MIS-C was a series of eight children seen at a tertiary centre in South East England. The most common comorbidities were obesity and asthma. The average age was 9–11 years old (range 1 to 17 years old) (10).

**Figure 4:**
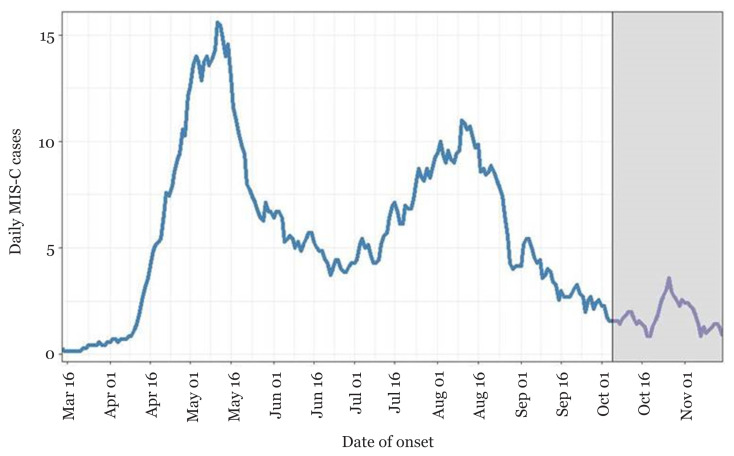
Daily MIS-C cases (7-day moving average) (25)

Based on the patterns seen in the UK, United States, France, Spain and Italy, there seems to be a lag of several weeks between the peak times of COVID-19 cases within communities to the peak of MIS-C cases (23). For example, in London, the peak time of COVID-19 cases occurred in the first to second weeks of April, while the peak of MIS-C cases occurred in the first to the second week of May (24). The incidence of cases in children in the United States has been gradually increasing from March to August 2020 (Figure 4). In the United States, MIS-C was diagnosed more in Black and Latino children as compared to other races and ethnic groups. Exploration is needed for determining why MIS-C affects these children more often than others. The discrepancies in access to health information and services besides the probability of risks associated with genetics could be considerable factors (25).

There is very little understanding of what triggers the MIS-C. It is of critical significance to collect the standardised data that describes the severity, clinical manifestations, outcomes and epidemiology of the disease (2). An initial case definition and case report form for MIS-C has been developed by the World Health Organization (WHO) for reflecting the clinical and laboratory findings identified in pediatric patients so far (5, 6). However, to have a deep understanding of MIS-C, there is a need for rigorous mapping of the disease and associated risk elements for better disease management and navigating through this crisis.

There is an immense requirement of increasing cognizance of this problem in the community and to follow WHO preventative measures. The best approach to preventing MIS-C is to minimise the contact of children with suspected or infected COVID-19 patients (17). Besides it is necessary to follow all the protective methods that include; repetitively washing hands for 20 sec as a minimum, avoiding individuals who are the sufferer of infectious disease, a distance of 6 feet between a child and another individual outside, wearing a mask, regular cleaning and disinfecting the touch surfaces regularly. The parents of a child with COVID-19 should be recommended to monitor the symptoms and to promptly notify the healthcare professionals; if they observe any specific indication.

## Conclusion

SARS-CoV-2 is a novel virus and only limited scientific evidence is available presently to comprehend its association with MIS-C. Though there has been a growing number of case reports and case series, the global and population-specific incidence of MIS-C remains unidentified, and the causal relationship and pathogenesis of KD and MIS-C remain indistinct. Even though there is some indication that the growth of MIS-C is a post-viral immunological reaction to COVID-19, recognising the immune response induced by SARS-CoV-2 remains poor. Clinical trials are required to find the optimal treatment; that perhaps reverse inflammatory processes and prevent coronary artery aneurysms. Future studies must explore whether the pathophysiology and mechanisms for the immune response of MIS-C will support the development of safe and effective SARS-CoV-2 vaccines for use in children. Lastly, international research collaboration is vital to provide potential treatment targets and develop the strategies for vaccines in a synchronised way.
